# Loss-of-Function Mutations in Three Homoeologous *PHYTOCLOCK 1* Genes in Common Wheat Are Associated with the Extra-Early Flowering Phenotype

**DOI:** 10.1371/journal.pone.0165618

**Published:** 2016-10-27

**Authors:** Nobuyuki Mizuno, Mika Kinoshita, Saki Kinoshita, Hidetaka Nishida, Masaya Fujita, Kenji Kato, Koji Murai, Shuhei Nasuda

**Affiliations:** 1 Laboratory of Plant Genetics, Graduate School of Agriculture, Kyoto University, Sakyo-ku, Kyoto, Japan; 2 Department of Bioscience, Fukui Prefectural University, Eiheiji-cho, Fukui, Japan; 3 Graduate School of Environmental and Life Science, Okayama University, Kita-ku, Okayama, Japan; 4 Institute of Crop Science, NARO, Tsukuba, Ibaraki, Japan; Institute of Genetics and Developmental Biology Chinese Academy of Sciences, CHINA

## Abstract

*Triticum aestivum* L. cv ‘Chogokuwase’ is an extra-early flowering common wheat cultivar that is insensitive to photoperiod conferred by the photoperiod insensitive alleles at the *Photoperiod-B1* (*Ppd-B1*) and *Ppd-D1*loci, and does not require vernalization for flowering. This reduced vernalization requirement is likely due to the spring habitat allele *Vrn-D1* at the *VERNALIZATION-D1* locus. Genotypes of the *Ppd-1* loci that determine photoperiod sensitivity do not fully explain the insensitivity to photoperiod seen in ‘Chogokuwase’. We detected altered expression patterns of clock and clock-output genes including *Ppd-1* in ‘Chogokuwase’ that were similar to those in an einkorn wheat mutant that lacks the clock-gene homologue, wheat *PHYTOCLOCK 1* (*WPCL1*). Presumptive loss-of-function mutations in all *WPCL1* homoeologous genes were found in ‘Chogokuwase’ and ‘Geurumil’, one of the parental cultivars. Segregation analysis of the two intervarietal F_2_ populations revealed that all the examined F_2_ plants that headed as early as ‘Chogokuwase’ had the loss-of-function *wpcl1* alleles at all three homoeoloci. Some F_2_ plants carrying the *wpcl1* alleles at three homoeoloci headed later than ‘Chogokuwase’, suggesting the presence of other loci influencing heading date. Flowering repressor *Vrn-2* was up-regulated in ‘Chogokuwase’ and ‘Geurumil’ that had the triple recessive *wpcl1* alleles. An elevated transcript abundance of *Vrn-2* could explain the observation that ‘Geurumil’ and some F_2_ plants carrying the three recessive *wpcl1* homeoealleles headed later than ‘Chogokuwase’. In spite of the up-regulation of *Vrn-2*, ‘Chogokuwase’ may have headed earlier due to unidentified earliness genes. Our observations indicated that loss-of-function mutations in the clock gene *wpcl1* are necessary but are not sufficient to explain the extra-early heading of ‘Chogokuwase’.

## Introduction

In temperate cereals such as barley and wheat, three factors, i.e., the vernalization requirement, photoperiod sensitivity and narrow-sense earliness (earliness *per se*) contribute to the timing of heading [1]. The vernalization requirement is controlled by three genes, namely, *VERNALIZATION 1* (*Vrn-1*), *Vrn-2* and *Vrn-3* [2]. *Vrn-1* encodes an *APETALA1*/*FRUITFUL*-like (*AP1/FUL*-like) MADS-box transcription factor [3–6]. Recessive alleles at all *Vrn-1* homoeoloci confer a winter growth habit (vernalization sensitive), whereas one or more dominant alleles at *Vrn-1* homoeoloci results in a spring growth habit (vernalization insensitive). The expression levels of *Vrn-1* in leaves are associated with earliness, suggesting that *Vrn-1* determines the flowering time [7]. Recently, *Vrn-D4* was identified as a duplicated copy of *Vrn-A1* [8]. The *Vrn-2* locus contains two tandemly duplicated genes (*ZCCT1* and *ZCCT2*) that encode a protein with a CCT (CONSTANS, CO-like, and TOC1) domain and a zinc-finger motif [9]. Non-functional mutations in the *ZCCT* genes result in a spring growth habit and, thus, early flowering [9–11], indicating their roles as flowering repressors. *Vrn-3* encodes a RAF kinase inhibitor-like protein with similarity to *Arabidopsis* FLOWERING LOCUS T (FT). Transgenic wheat plants overexpressing *Vrn-3* have an extra-early flowering phenotype without the need for vernalization [12–13], indicating that *Vrn-3* is a strong flowering promoter. Hereafter, we denote *Vrn-3* as *WFT* (wheat *FT*). Three models, although controversial, for the epistatic interaction between these vernalization genes have been proposed [13–16].

Photoperiod insensitive alleles of *Ppd-1* (*Ppd-1a*) were identified for each homoeolocus on chromosomes 2A, 2B and 2D of common wheat, respectively [17–20]. Three alleles of *Ppd-A1a* and one allele of *Ppd-D1a* possess deletions in a shared promoter region [18–19, 21]. The cultivars carrying these *Ppd-1a* alleles had an increased expression level with an abnormal circadian rhythm for *Ppd-1* expression. Novel mutations were found in the 5’ upstream regions of *Ppd-A1* and *Ppd-B1* [21]. In addition to the mutations in the promoter region, increased copy numbers of *Ppd-B1* altered its expression pattern and accelerated flowering time [22–23]. Wheat plants with at least one *Ppd-1a* allele were associated with an increased expression level of *TaFT* (*WFT*), which correlates with the early flowering phenotypes [20]. In barley and wheat, the orthologues of circadian clock genes in *Arabidopsis* have been identified as candidate genes conferring the early flowering phenotype [24–28]. Recently, we showed that a wheat homologue of *Arabidopsis LUX ARRHYTHMO*/*PHYTOCLOCK 1 (LUX/PCL1)*, which is located on the long arm of wheat chromosome 3A, is a candidate gene that is missing in an early flowering mutant of einkorn wheat (*Triticum monococcum* L.) [25]. In *Arabidopsis*, LUX/PCL1 constitutes the Evening Complex (EC) together with EARLY FLOWERING 3 (ELF3) and ELF4 [29]; the EC directly represses *PRR9* [30]. The barley and wheat mutants of *LUX*/P*CL1* and *ELF3* homologues head earlier than the wild-type lines under both long day and short day conditions [24–28]. In the einkorn wheat mutant lacking the wheat *LUX*/*PCL1* homologue (*WPCL1*), *Ppd-1* and *WFT* were up-regulated, whereas *LATE ELONGATED HYPOCOTYL* (*LHY*) and *TIMING OF CAB EXPRESSION 1 (TOC1*) were down-regulated [25, 28]. These expression patterns were also observed in the *Arabidopsis lux* mutants [31]. These results strongly suggest that barley and wheat LUX/PCL1 function similarly to their counterparts in *Arabidopsis*.

Intermittent rain before harvest often causes pre-harvest sprouting and poor grain quality in wheat cultivated in East Asia [32]. To avoid these problems, early-heading wheat cultivars have been bred in many countries. Among 260 wheat cultivars examined, ‘Chogokuwase’, a super-early-heading wheat cultivar in Japan, is one of the earliest heading cultivars [33]. The heading time for ‘Chogokuwase’ was much earlier than the parental cultivars, ‘Minaminokomugi’ and ‘Geurumil’ (Table 1), suggesting that earliness genes were inherited from both parents. Seki et al. (2011, 2013) [33–34] showed that ‘Chogokuwase’ had the *Ppd-D1a* allele and there are no differences in the *Ppd-1* genotypes between ‘Chogokuwase’ and the parental cultivars at any of the homoeoloci. This result strongly suggests that genes other than *Ppd-1* contribute to the earliness of ‘Chogokuwase’. Here, we conducted gene expression analyses, sequence analyses and segregation analyses to elucidate the reasons for earliness in ‘Chogokuwase’. Our results indicate that loss-of-function alleles of *WPCL1* homoeologues contribute to the earliness of ‘Chogokuwase’. We discuss the function of *WPCL1* in the photoperiod and vernalization pathways of the wheat flowering gene network.

**Table 1:** Heading dates of ‘Chogokuwase’ and the parental cultivars in the field.

Cultivar	2011/2012^a^	2012/2013^a^
‘Chogokuwase’	18 March	24 March
‘Minaminokomugi’	23 April	15 April
‘Geurumil’	28 April	23 April

^a^ Sowing dates were 19 Oct 2011 or 17 Oct 2012, respectively.

## Materials and Methods

### Plant materials

‘Chogokuwase’ is an offspring of a cross between a Japanese cultivar ‘Minaminokomugi’ and a Korean cultivar ‘Geurumil’. The common wheat cultivar ‘Chinese Spring’ (CS) was used to determine copy number variation and for sequence analyses. We used the following two F_2_ populations for segregation analyses: (1) the “CN population” consisted of 114 F_2_ plants derived from a cross between ‘Chogokuwase’ and ‘Norin 61’, and (2) the “MG population” consisted of 489 F_2_ plants derived from a cross between ‘Minaminokomugi’ and ‘Geurumil’. Recombinant inbred lines (F_11_ generation) that were derived from the cross between cultivated einkorn wheat *T*. *monococcum* L. (KT3-5) and wild einkorn wheat *T*. *boeoticum* Boiss. (KT1-1) were used for an analysis of epistatic interactions between *Vrn-2* and *WPCL1* [25].

### Field experiments

‘Chogokuwase’, ‘Minaminokomugi’ and ‘Geurumil’ were sown in the middle of October in an experimental field (36.108 N, 136.275 E) at Fukui Prefectural University, Fukui, Japan (2011/2012 and 2012/2013 seasons). Sowing dates were 19 Oct 2011 and 17 Oct 2012. The heading dates of each cultivar were recorded.

### Measurement of heading time in a growth chamber

To investigate their photoperiod sensitivity and vernalization requirement, plants were cultivated in a growth chamber under long-day (16 h light and 8 h dark; LD) or short-day (10 h light and 14 h dark; SD) conditions at 20°C (light intensity ~100 μE m^-2^ s^-1^). Seedlings at 5-days after germination were subjected to a vernalization treatment (5°C) for 21 days. After the vernalization treatment, plants were grown at 20°C. Heading time was measured in at least three replicated samples as the number of days between unfolding of the third leaf and the flag leaf. Differences between samples were statistically examined by Tukey Kramer’s HSD tests (*P* < 0.05).

### Genotyping of *Vrn-1* and *Ppd-1* homoeoloci

We used polymerase chain reaction (PCR) primers (S1 Table) that had been shown to identify the alleles of *Vrn-1* homoeoloci in previous studies [35–36]. Genotypes of the three *Ppd-1* homoeoloci in ‘Chogokuwase’, ‘Minaminokomugi’, ‘Geurumil’ and ‘Norin 61’ were determined as described in Seki et al. (2011, 2013) [33–34]. To estimate the copy number of the *Ppd-B1* loci, real-time PCR analyses were carried out using a LightCycler Nano System (Roche Diagnostics, Switzerland). A wheat *CONSTANS2* gene, *TaCO2* (also called *TaHd-1*), was used as the internal control. We used gene-specific primer sets for *Ppd-B1* and *TaCO2* as reported in a previous report [22]. The rate of amplification was monitored using THUNDERBIRD SYBR qPCR mix (TOYOBO, Japan) according to the manufacturer’s protocol. Amplification rates of the samples in five technical replications were converted to copy numbers by comparing the rates in common wheat cultivars ‘Cheyenne’, ‘Timstein’ and ‘CS’ that are known to possess one, three, and four copies of *Ppd-B1*, respectively [22].

### Quantitative reverse-transcriptase (RT)-PCR

Expression of *Vrn-1* and *WFT* was analyzed in non-vernalized plants, 21 days-vernalized plants grown under the LD conditions, and in 21 days-vernalized plants grown under the SD conditions. For each cultivar, seedlings were sampled from the 2-leaf stage to the 4-leaf stage. In this study, the 1-leaf stage was defined as the period from the unfolding of the first leaf to the unfolding of the second leaf. Total RNAs were extracted from leaves using ISOGEN (Nippon Gene, Japan). cDNAs were synthesized from the total RNAs using an oligo dT primer according to the protocol for the Ready-To-Go T-primed First-Strand Kit (GE Healthcare Life Sciences, USA). Real-time PCR analyses were performed in three technical replications using a LightCycler 2.0 (Roche Diagnostic, Switzerland) with the gene-specific primer sets shown in Table 2. Transcript abundance was determined relative to a SYBR Green-labelled amplification product of the wheat *Actin* gene (AB1819991) that is often used as an internal control for gene-expression analyses under various conditions [25, 37].

**Table 2:** Alleles at the *Ppd-1* and *Vrn-1* loci.

Cultivar	*Ppd-1*^a^	*Vrn-1*^b^	*WPCL1*
‘Chogokuwase’	*Ppd-A1b/Ppd-B1b/Ppd-D1a*	*vrn-A1/vrn-B1/VRN-D1*	*wpcl-A1*/*wpcl-B1*/*wpcl-D1*
‘Minaminokomugi’	*Ppd-A1b/Ppd-B1b/Ppd-D1a*	*vrn-A1/vrn-B1/VRN-D1*	*wpcl-A1*/*WPCL-B1*/*WPCL-D1*
‘Geurumil’	*Ppd-A1b/Ppd-B1b/Ppd-D1a*	*vrn-A1/vrn-B1/vrn-D1*	*wpcl-A1*/*wpcl-B1*/*wpcl-D1*
‘Norin 61’	*Ppd-A1b/Ppd-B1b/Ppd-D1a*	*vrn-A1/vrn-B1/VRN-D1*	*WPCL-A1*/*WPCL-B1*/*WPCL-D1*

^a^ Alleles designated according to Seki et al. 2011 [33] and 2013 [34]

^b^ In the absence of dominant *VRN-A1* and *VRN-B1* alleles, the presence of a dominant *VRN-D1* confers a spring habit to ‘Chogokuwase’ and ‘Minaminokomugi’, and a recessive *vrn-D1* confers a winter habit to ‘Geurumil’.

For the expression analysis of the clock and clock-output genes, plants were grown at 23°C under SD conditions (9 h light and 15 h dark). Two-week-old leaves were sampled every three hours. Total RNA was isolated using an RNeasy Plant Mini Kit (Qiagen, Germany). First-strand cDNA was synthesized from 1 μg RNA in a 20 μL reaction solution with oligo-dT primers using a ReverTra Ace -α- Kit (TOYOBO, Japan). Primers used for quantitative RT-PCR are shown in S2 Table. Relative transcript levels were determined in three technical replications using a LightCycler Nano System with FastStart Essential DNA Green Master (Roche Diagnostics, Switzerland). Quantitative RT-PCR was performed according to the manufacturer’s protocol using the wheat *Actin* gene as the internal control.

### Sequencing of three *WPCL1* homoeologues

Total DNA was extracted from leaves of common wheat using a DNeasy Plant Mini Kit (Qiagen, Germany). To obtain genome-specific primers, we searched for the sequences of all three *WPCL1* homoeologues in the chromosome-arm specific survey sequences of 3AL, 3B and 3DL [38]. First, a blastn search against the survey sequences was performed using the *WPCL1* sequences of *T*. *monococcum* L. (accession number; AB773826) [25] as a query. Alignments of the survey sequences obtained from the three genomes led to the design of the following genome-specific primers to amplify the entire coding region: *WPCL-A1*; 5′-GCTCCAAAATGGGTCCAGAGGA-3′ and 5′- GGACAGTGAGTCCCAAATCTGA -3′, *WPCL-B1*, 5′- TGCGCAAATTAAATATCCGACAGA -3′ and 5′- GATCGACACAAACACACGCC -3′; *WPCL-D1*, 5′- ATCTATCCACCATCCATGCG-3′ and 5′- CCGGACAGGACACATTCACA -3′. Thirty-three cycles of PCR were performed using KAPATaq Extra (Kapa Biosystems, USA) and the following reaction conditions: 30 s at 94°C, 30 s at 60°C, and 4 m at 72°C. Nucleotide sequences were determined with BigDye Terminator version 3.1 (Applied Biosystems, USA) using an Applied Biosystems 3730xl DNA Analyzer. Nucleotide sequences and their predicted amino acid sequences were analyzed by GENETYX-MAC version 12.00 software (Whitehead Institute for Biomedical Research, USA).

### Segregation analyses

One hundred fourteen F_2_ plants (CN population) from a cross between ‘Chogokuwase’ and ‘Norin 61’ were used in the first segregation analysis. The plants were grown at 23°C for ten days and then vernalized at 4°C for seven weeks. After vernalization, the plants were incubated at 23°C and grown under SD conditions (8 h light and 16 h dark). In addition to the CN population, the segregation of 489 F_2_ plants (MG population) from a cross between ‘Minaminokomugi’ and ‘Geurumil’ was also analyzed. The heading dates of the F_2_ plants in the MG population were determined in the experimental field at Fukui Prefectural University, Fukui, Japan in the 2013/2014 cultivation season. For genotyping of *WPCL-A1* and *WPCL-D1*, PCR was carried out using following primer sets: *WPCL-A1*; 5′-CCACGCCAACGGCGGC-3′ and 5′-TGAATCCGGGATGGATGGTTATC-3′, *WPCL-D1*; 5′-CGGCGGCTGGGGATGAC-3′ and 5′-TTGATGACTGAACTGGACCGATCT-3′. For genotyping of *WPCL-B1*, multiplex PCR was conducted using the following sets of primers: 5′-TCGGATTGGTGTTGCGAGG-3′ and 5′-CATGATGGTCTTGGGCACC-3′, and 5′-ACGTAGTACTCCCTTGGTCC-3′ and 5′-CAACCGAACCTATGAACACCA-3′. Thirty-four cycles of PCR were performed using KAPATaq Extra (Kapa Biosystems, USA) and following reaction conditions: 30 s at 94°C, 30 s at 60°C, and 45 s at 72°C. To detect allelic variation, the PCR products of *WPCL-A1* and *WPCL-D1* were treated with *Rsa*I and *Bsp*T*107*I, respectively. For genotyping of *Vrn-D1*, two primer sets (Intr1/D/F- Intr1/D/R3 and -Intr1/D/R4) were used according to Yan et al. (2004) [35]. The amplified PCR products were separated by electrophoresis through a 1.0% or 1.5% agarose gel and stained with ethidium bromide.

## Results

### Reduced vernalization requirement and photoperiod sensitivity in ‘Chogokuwase’

**Fig 1:**
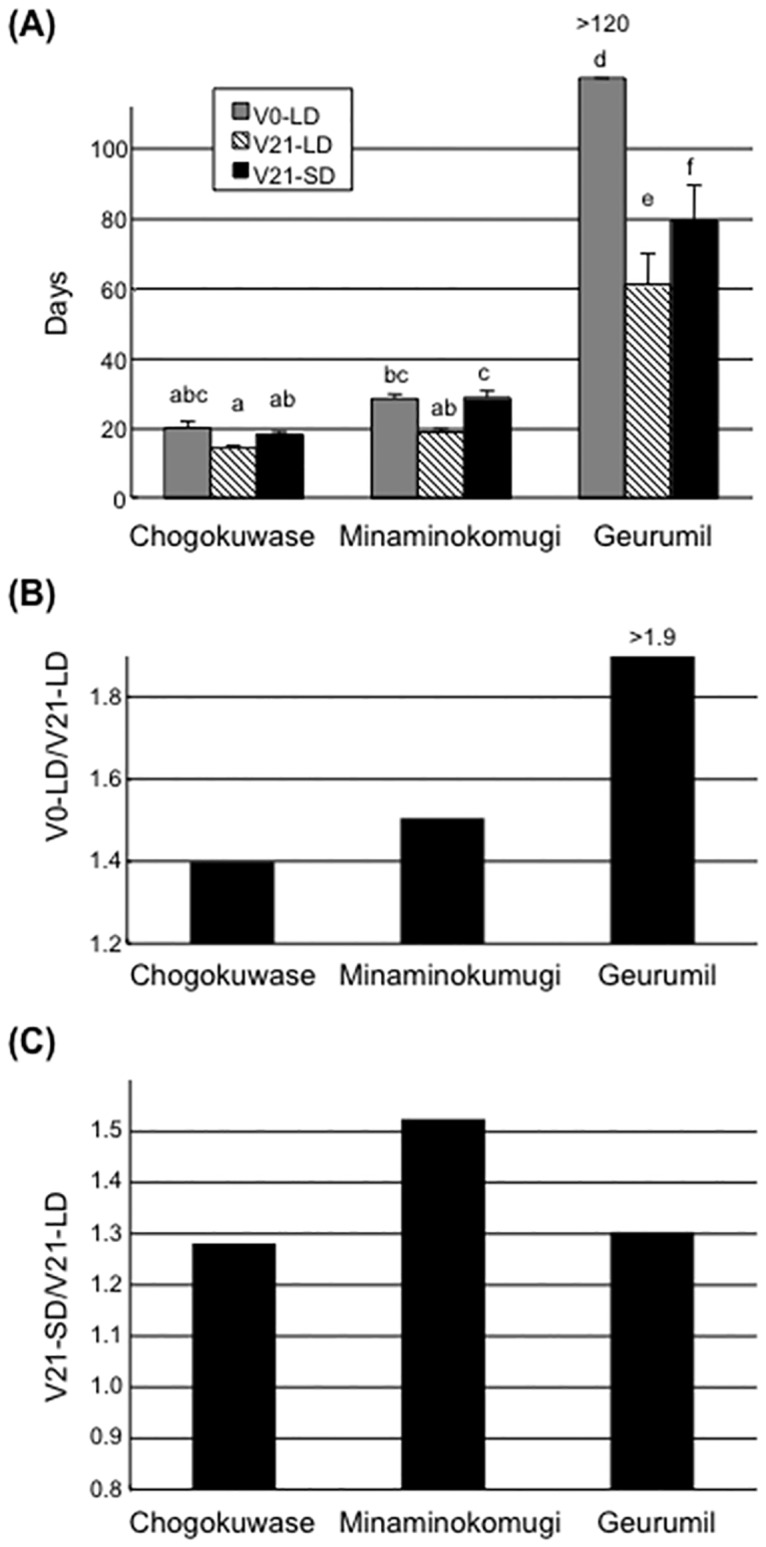
Comparison of days to heading for ‘Chogokuwase’ and its parental cultivars as measured by unfolding of the flag leaf. (a) The days to unfolding of the flag leaf without vernalization treatment under long day conditions (V0-LD), after 21-days vernalization under long days (V21-LD) and after 21-days vernalization under short days (V21-SD). The different letters over the bars indicate statistically significant differences by Tukey Kramer’s HSD test (*P* < 0.05). (b) Comparison of the vernalization requirement among three cultivars as indicated by the ratio of V0-LD over V21-LD. (c) Comparison of the photoperiod sensitivity as represented by the ratio of V21-SD over V21-LD for the three cultivars.

‘Chogokuwase’ headed more than three weeks earlier than the parental cultivars ‘Minaminokomugi’ and ‘Geurumil’ in the field (Table 1). To investigate the vernalization requirement and photoperiod sensitivity of ‘Chogokuwase’, heading dates of ‘Chogokuwase’, ‘Minaminokomugi’ and ‘Geurumil’ were recorded under LD conditions without vernalization (V0-LD), LD conditions with 21 days vernalization (V21-LD), and SD conditions with 21 days vernalization (V21-SD). In all conditions, ‘Chogokuwase’ headed earlier than ‘Geurumil’ (Fig 1, panel (a)). ‘Chogokuwase’ had a reduced vernalization requirement compared with ‘Geurumil’, and the vernalization requirement of ‘Chogokuwase’ was as low as that of ‘Minaminokomugi’ (Fig 1, panel (b)). The photoperiod sensitivity of ‘Chogokuwase’ was similar to but lower than that of ‘Minaminokomugi’ (Fig 1, panel (c)).

### ‘Chogokuwase’ has a spring habit allele for *VRN-D1* and an increased number of *Ppd-B1* copies

*Vrn-1* and *Ppd-1* are the major genes that determine the vernalization requirement and photoperiod sensitivity, respectively. PCR analysis revealed that ‘Chogokuwase’ and ‘Minaminokomugi’ carried a *Vrn-D1* spring habit allele, whereas ‘Geurumil’ had a winter habit *vrn-D1* (Table 2). The alleles of *Vrn-A1* and *Vrn-B1* loci in these three cultivars were winter habit types. The three cultivars had a photoperiod insensitive *Ppd-D1a* allele and photoperiod sensitive *Ppd-A1b* and *Ppd-B1b* alleles [33–34]. The photoperiod insensitive *Ppd-B1a* allele is associated with a higher number (2 to 4) of copies of *Ppd-B1* [22–23]. Copy number estimation revealed that ‘Chogokuwase’ and ‘Minaminokomugi’ had three *Ppd-B1* copies (S1 Fig) and an additional truncated copy (data not shown) as was reported for a Nepalese cultivar by Nguyen et al. (2013) [23].

### Elevated transcript levels of *Vrn-1* and *WFT* in ‘Chogokuwase’

**Fig 2:**
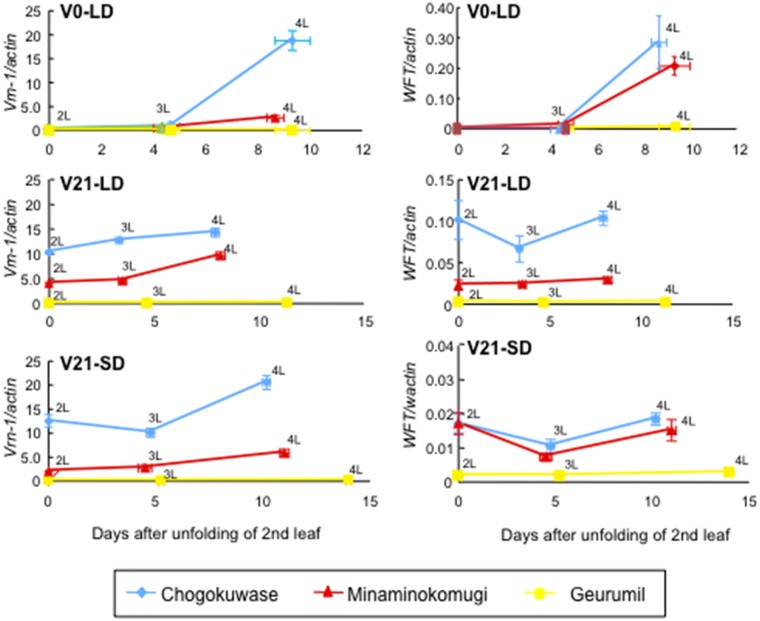
Gene expression patterns of *VRN-1* and *WFT* in ‘Chogokuwase’ and its parental cultivars. Expression levels were measured in the second (2L), third (3L) and fourth (4L) leaf stages of ‘Chogokuwase’ (blue), ‘Minaminokomugi’ (red), and ‘Geurumil’ (yellow) under V0-LD, V21-LD and V0-SD conditions. X- and Y-axis indicate days after 21 days-vernalization and relative transcript abundances, respectively. Transcript abundance was measured relative to the abundance of *Actin* gene transcripts. Bars on each observation indicate standard errors.

Since the transcript abundance of *Vrn-1* and *WFT* are often correlated with heading time in wheat [7], we examined the expression of these genes in ‘Chogokuwase’ and its parental cultivars. *Vrn-1* transcript levels were significantly higher in ‘Chogokuwase’ than in the parental cultivars at all 4L stages under V0-LD, V21-LD and V21-SD conditions (Fig 2). Levels of *Vrn-1* transcripts in ‘Geurumil’ were lower than in ‘Minaminokomugi’. *WFT* expression was up-regulated in ‘Chogokuwase’ especially under V21-LD conditions. Under the V0-LD and V21-SD condition, however, obvious difference was not observed in *WFT* transcript abundance between ‘Chogokuwase’ and ‘Minaminokomugi’. *Vrn-1* transcript abundance was associated with the heading time rather than that of *WFT*.

### Expression patterns of circadian clock and clock-output genes were altered in ‘Chogokuwase’

**Fig 3:**
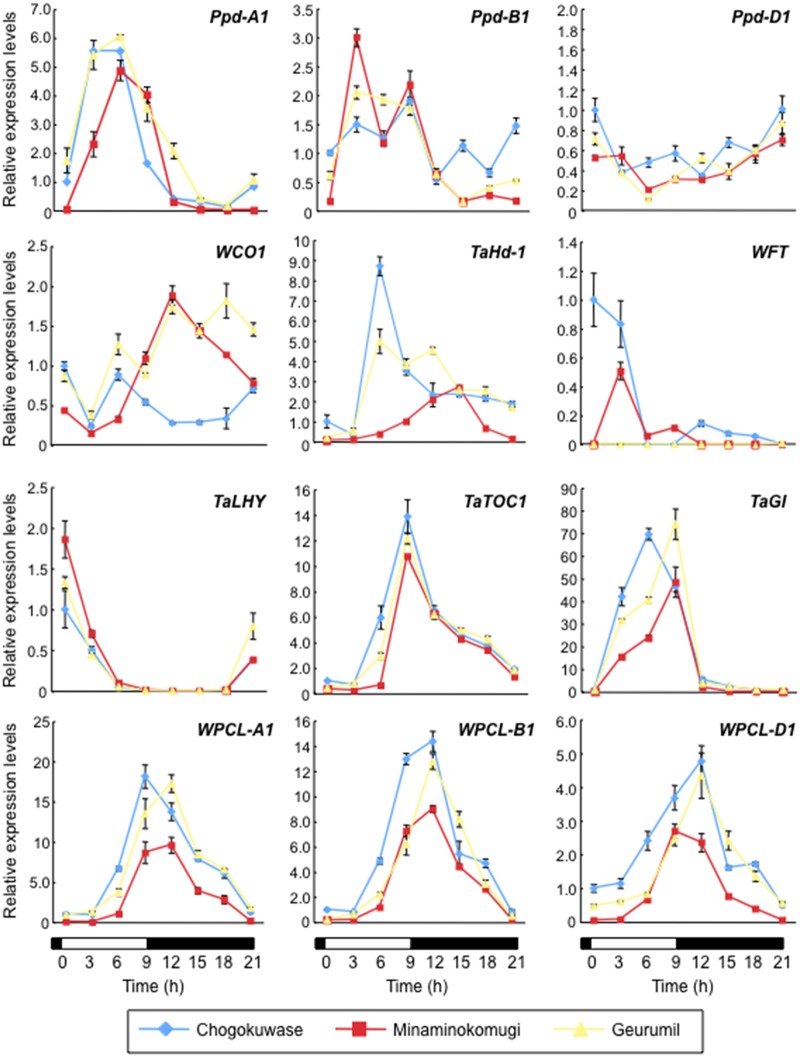
Gene expression patterns of circadian clock and clock-output genes under SD conditions in ‘Chogokuwase’ and the parental cultivars. White and black boxes indicate light and dark periods, respectively. Two-week-old seedlings were sampled every 3 hours over 24 h. Means ± standard deviations were calculated from data in three technical repeated experiments. Relative transcript abundance was calculated using the *Actin* gene as an internal control.

Diurnal expression patterns of *Ppd-1*, two wheat *CONSTANS*-like genes (*WCO1* and *TaHd1*) and *WFT* were examined under SD conditions by quantitative RT-PCR. The transcript accumulation levels of *Ppd-A1* in the early light period were higher in ‘Chogokuwase’ and ‘Geurumil’ than in ‘Minaminokomugi’ (Fig 3). A higher expression level of *Ppd-B1* was observed in ‘Chogokuwase’ in the late dark period. Unlike *Ppd-A1* and *Ppd-B1*, *Ppd-D1* expression levels fluctuated, and no obvious differences in patterns among the three cultivars were evident. In ‘Chogokuwase’, the expression level of *WCO1* during the dark period was low, whereas *TaHd-1* was up-regulated in ‘Chogokuwase’ and ‘Geurumil’ compared with ‘Minaminokomugi’. The expression level of *TaLHY*, a clock gene, in ‘Chogokuwase’ and ‘Geurumil’ was significantly lower than ‘Minaminokomugi’ at the peak of its expression (beginning of the day). In ‘Chogokuwase’ and ‘Geurumil’, on the other hand, transcripts of another clock gene *TaTOC1* accumulated earlier than in ‘Minaminokomugi’. This early accumulation pattern was more evident for the third clock gene *GIGANTEA* (*TaGI*).

### Sequence analysis of *WPCL1*

**Fig 4:**
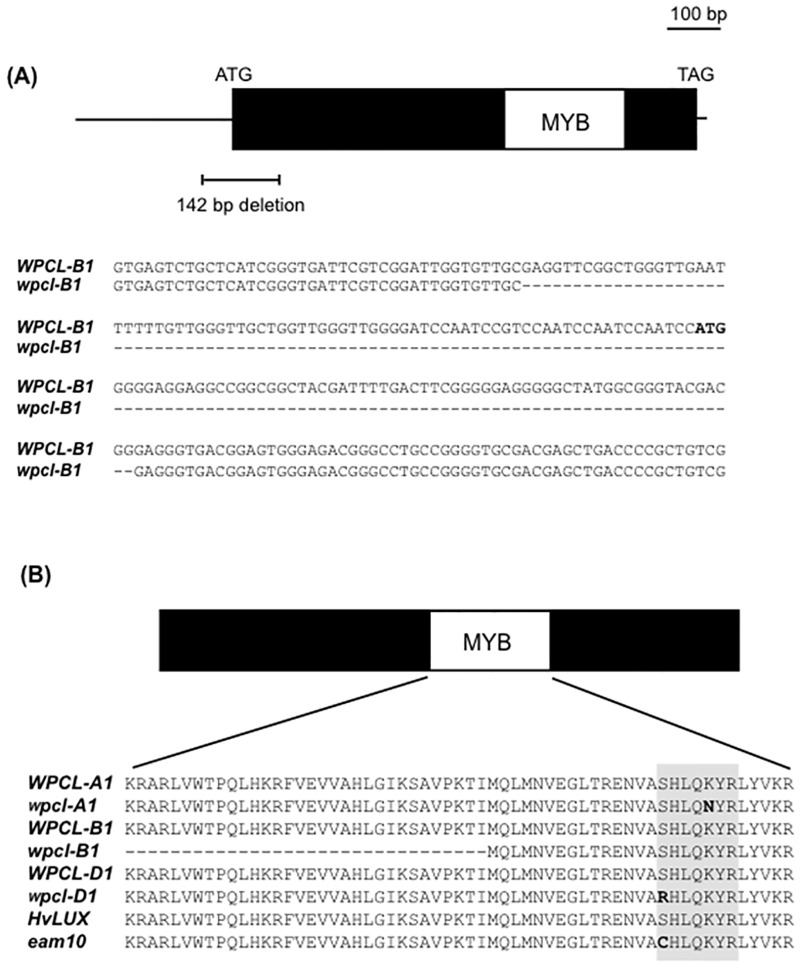
Mutations in *WPCL1* homoeologues. (a) A 142 bp deletion was detected in *WPCL-B1* of ‘Chogokuwase’ and ‘Geurumil’. The start codon is shown in bold letters in the nucleotide sequence alignment. (b) Comparison of the amino acid sequence of the Myb domain between functional (gene names shown in uppercase letters) and loss-of-function (gene names shown in lowercase letters) *WPCL1* homoeologues. The gray box indicates the SHAQKYF motif.

The expression patterns of clock and clock-output genes in ‘Chogokuwase’ and ‘Geurumil’ are similar to those in the einkorn wheat mutant lacking *WPCL1* [25]. We determined the nucleotide sequences of *WPCL-A1*, *WPCL-B1* and *WPCL-D1* in ‘Chogokuwase’ and the parental cultivars. A 142 bp deletion was observed in the region spanning the 5′ untranslated region (UTR) and the first exon of *WPCL-B1* in ‘Chogokuwase’ and ‘Geurumil’ (Fig 4, panel (a)). The deletion in the first exon was 65 bp that would result in a frameshift mutation. We did not find proper reading frames in *WPCL-B1* of ‘Chogokuwase’ and ‘Geurumil’, although we sequenced 900 bp upstream of the translation start codon. The longest presumed open reading frame of *WPCL-B1* was 363 bp that could be translated into a truncated protein lacking half of the Myb domain (Fig 4, panel (b)), suggesting that this allele of *WPCL-B1* in ‘Chogokuwase’ and ‘Geurumil’ was loss-of-function (*wpcl-B1*). Furthermore, a single nucleotide polymorphism (SNP) was found in *WPCL-D1* of ‘Chogokuwase’ and ‘Geurumil’ in the SHAQKYF motif, a highly conserved region in the MYB domain [27] and whose consensus sequence in plant *LUX*-like genes is SHLQKY(R/Q). ‘Minaminokomugi’ and ‘CS’ had the SHLQKYR motif in *WPCL-D1*. The SNPs altered the amino acid sequence of this motif to CHLQKYR in *WPCL-D1* of ‘Chogokuwase’ and ‘Geurumil’. ‘Chogokuwase’, ‘Minaminokomugi’ and ‘Geurumil’ shared another non-synonymous (K to N) substitution within the amino acid sequences of the SHAQKYF motif in *WPCL-A1*. The SHAQKYF motif is highly conserved in functional MYB transcription factors, and an amino acid substitution in this motif was associated with a loss-of-function mutation in the barley *LUX*/*PCL1* gene *HvLUX1* [27]. Thus, we assumed that the *wpcl-A1* and *wpcl-D1* alleles found in ‘Chogokuwase’ and ‘Geurumil’ were loss-of-function. In fact, the expression levels of *wpcl-A1*, *wpcl-B1* and *wpcl-D1* were consistently higher in ‘Chogokuwase’ and ‘Geurumil’ than in ‘Minaminokomugi’ (Fig 3). This finding is similar to that reported for the loss-of-function *lux*/*pcl1* mutants in *Arabidopsis* and barley where *LUX*/*PCL1* was up-regulated due to a feedback loop [27, 30].

### Plants homozygous for the *wpcl* allele at three homoeoloci had an early heading phenotype

**Fig 5:**
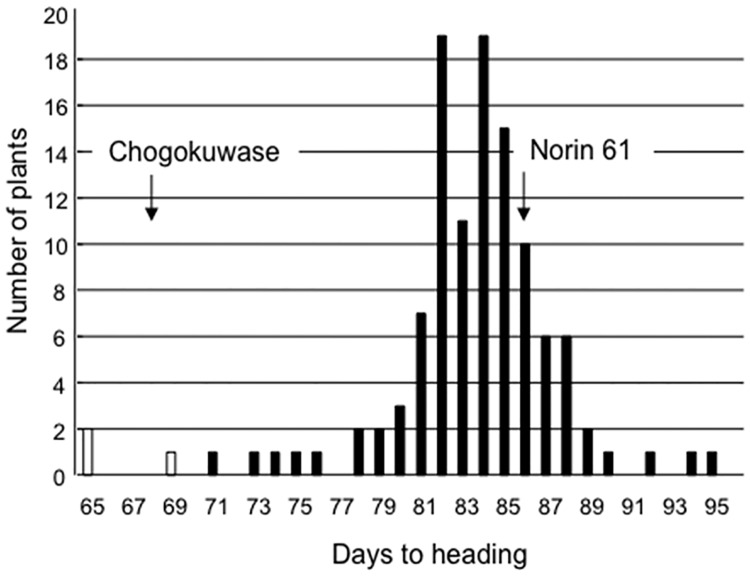
Frequency distribution of days to heading in the 114 F_2_ plants from a segregating population growing under SD conditions with a vernalization treatment. The F_2_ population was derived from a cross between ‘Chogokuwase’ and ‘Norin 61’. White boxes indicate the plants with homozygous *wpcl1* alleles at three homoeoloci. Black boxes show the plants having one or more functional *WPCL1* homoeoalleles. Arrows indicate days to heading in ‘Chogokuwase’ and ‘Norin 61’, respectively.

**Fig 6:**
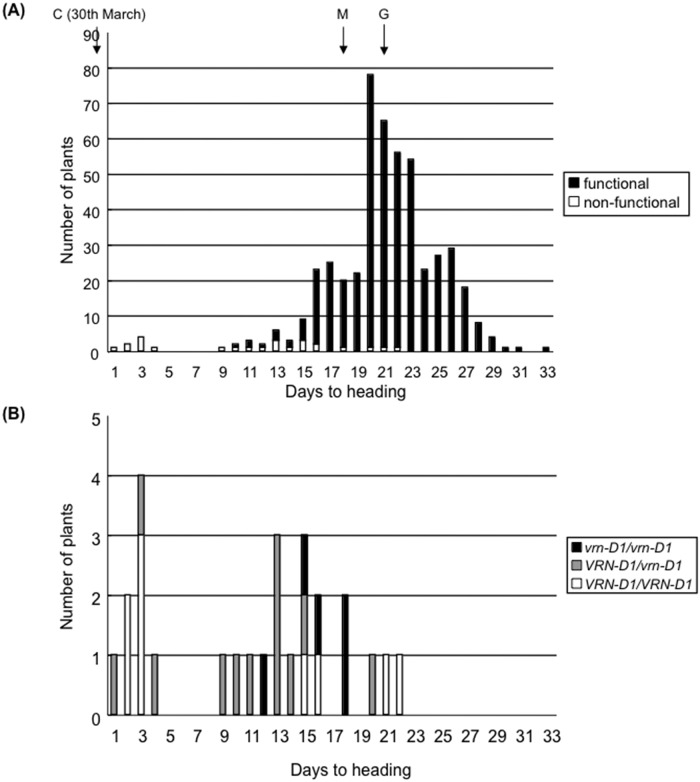
Relationship between days to heading and genotypes of *WPCL1* and *Vrn-1* loci in an F_2_ population derived from ‘Geurumil’ and ‘Minaminokomugi’. (a) Comparison of days to heading between genotypes of *WPCL1* homoeoloci in the 489 F_2_ plants. Days to heading indicate the number of days after April 1. Arrows show the heading date in ‘Chogokuwase’ (C), ‘Geurumil’ (G) and ‘Minaminokomugi’ (M), respectively. White boxes indicate the plants carrying homozygous *wpcl1* alleles at three homoeoloci. Black boxes show the plants carrying functional *WPCL-B1* and*/*or *WPCL-D1* alleles. (b) A subset of the segregating population having the triple recessive *wpcl1* alleles. Comparison of days to heading between genotypes of the *Vrn-D1* locus in 25 F_2_ plants with three loss-of-function *wpcl1* homoeoalleles.

Segregation analyses in two F_2_ populations segregating the *WPCL1* loci (CN and MG populations) were used to test the association between the early heading phenotype and genotypes at the *WPCL1* homoeoloci. In the CN population derived from a cross between ‘Chogokuwase’ (all loss-of-function *wpcl1*) and ‘Norin 61’ (all functional *WPCL1*), a triple recessive individual should appear in one out of 64 plants and individuals in the population should be homozygous at all *Ppd-1* and *Vrn-1* homoeoloci (Table 2, S1 Fig). Of the 114 F_2_ plants, only three plants carrying homozygous *wpcl-A1*, *wpcl-B1* and *wpcl-D1* alleles headed as early as ‘Chogokuwase’ (Fig 5). The segregation ratio fit a 1:63 ratio (*χ*^2^ = 0.85, *P* = 0.36). The other 111 F_2_ plants were homozygous for a dominant allele or heterozygous at the *WPCL1* locus and headed later than the three triple recessive plants. In the MG population, only alleles at the *WPCL-B1* and *WPCL-D1* loci segregated because ‘Geurumil’ and ‘Minaminokomugi’ have the *wpcl-a1* allele. Of the 489 F_2_ plants, 26 plants carrying the homozygous *wpcl1* at the *WPCL-B1* and *WPCL-D1* loci were found. Of the 26 *wpcl1* plants, eight plants headed as early as ‘Chogokuwase’ (Fig 6, panel (a)). The segregation ratio for early (n = 8) and late (n = 18) heading fit a 1:3 ratio (*χ*^2^ = 0.462, *P* = 0.50), but also a 3:13 ratio (*χ*^2^ = 2.47, *P* = 0.12), which does not rule out the presence of other loci controlling heading. We found that all the early heading plants were homozygous for the triple recessive *wpcl1* homoeologues, and were vernalization-insensitive due to presence of dominant *VRN-D1* allele (Fig 6, panel; (b)). In the *wpcl1* triple recessive genetic background, the effect of copy-number variation at the *Ppd-B1* locus on early heading is negligible as indicated by the segregation of the truncated copy of *Ppd-B1* in five of the eight early-heading plants (data not shown).

### Epistatic interactions between *WPCL1* and vernalization genes

**Fig 7:**
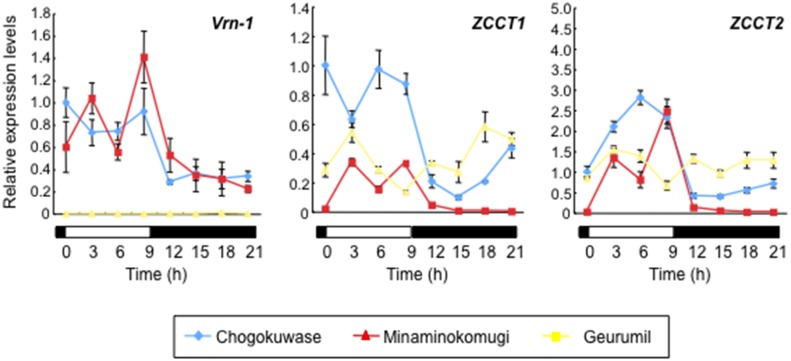
Gene expression patterns of *Vrn-1* and *Vrn-2* (*ZCCT1* and *ZCTT2*) under SD conditions in ‘Chogokuwase’ and the parental cultivars. White and black boxes indicate light and dark periods, respectively. Two-week-old seedlings were sampled at 3h intervals over 24h. Means ± standard deviations were calculated from data in three technical repeated experiments. Relative transcript abundance was calculated using the *Actin* gene as an internal control.

Day-neutral mutations in wheat *WPCL1* and barley *ELF3* up-regulate *Vrn-2* expression, resulting in a stronger vernalization requirement [39]. Our quantitative RT-PCR experiment demonstrated that the *Vrn-2* genes (*ZCCT1* and *ZCCT2*) were expressed in ‘Chogokuwase’, ‘Minaminokomugi’ and ‘Geurumil’ (Fig 7), although *Vrn-2* is generally not expressed under SD conditions [40–41]. Transcript abundance of *Vrn-2* in ‘Chogokuwase’ and ‘Geurumil’ was higher than that in ‘Minaminokomugi’ during dark period. The expression level of *Vrn-1* in the *vrn-D1* allele carrier ‘Geurumil’ (Table 2) was extremely low, although *Vrn-2* expression was comparable to that in ‘Chogokuwase’. These results suggest that the expression levels of *Vrn-1* were determined by the genotype of the *Vrn-D1* locus rather than by the expression levels of *Vrn-2*.

**Fig 8:**
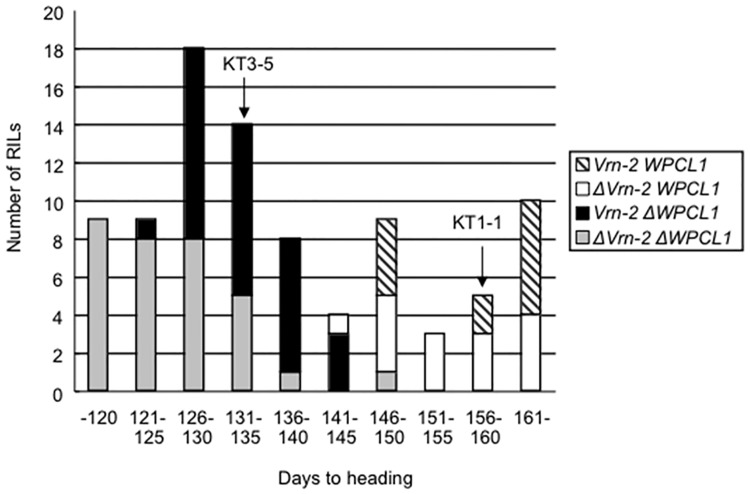
Relationship between days to heading and genotypes of the *WPCL1* and *Vrn-2* loci in the recombinant inbred lines of einkorn wheat. The days to heading in 89 recombinant inbred lines derived from a cross between *T*. *monococcum* mutants and *T*. *boeoticum* were compared. The parental *monococcum* accession lacks *WPCL1* and *Vrn-2*, whereas the *boeoticum* accession has *WPCL1* and *Vrn*-2. Arrows indicate days to heading of the parental *T*. *monococcum* (KT3-5) and *T*. *boeoticum* (KT1-1) accessions, respectively.

The recombinant inbred lines (RILs) resulting from a cross between a *T*. *boeoticum* accession with *WPCL1* and *Vrn-2* and an einkorn wheat mutant with deletions at both loci (denoted as Δ*WPCL1* and Δ*Vrn-2*) [25] made it possible to test epistatic interactions between *WPCL1* and *Vrn-2*. Of the 62 RILs with Δ*WPCL1*, the RILs with Δ*Vrn-2* headed significantly earlier than those carrying *Vrn-2* (Fig 8; *P* < 0.001). In contrast, among the RILs carrying *WPCL1*, there was no significant difference in heading time between the RILs with and without *Vrn-2*.

## Discussion

### ‘Chogokuwase’ and ‘Geurumil’ contain loss-of-function natural variants of the *WPCL1* gene

‘Chogokuwase’ is an extra-early heading cultivar that was derived from ‘Minaminokomugi’ and ‘Geurumil’ [33] and has a reduced vernalization requirement and lower sensitivity to photoperiod (Fig 1). The reduced vernalization requirement and photoperiod sensitivity of ‘Chogokuwase’ were derived from the parental cultivars ‘Minaminokomugi’ and ‘Geurumil’, respectively. *Vrn-1* and *Ppd-1* are major genes determining the vernalization requirement and photoperiod sensitivity in common wheat [6, 17]. The vernalization insensitivity of ‘Chogokuwase’ could be explained by the dominant allele of *Vrn-D1* that was inherited from ‘Minaminokomugi’. In contrast, *Ppd-1* cannot be a candidate gene for the reduced photoperiod sensitivity of ‘Chogokuwase’ because ‘Minaminokomugi’ and ‘Chogokuwase’ have the same genotypes for the three *Ppd-1* homoeoloci (Table 2, S1 Fig). The expression patterns of clock and clock-output genes in ‘Chogokuwase’ and ‘Geurumil’ were similar to those of an einkorn wheat mutant that lacks *WPCL1* [25] and were different from those in ‘Minaminokomugi’. Sequence analyses revealed that ‘Chogokuwase’ and ‘Geurumil’ had non-synonymous substitutions in the SHAQKYF motif of both *WPCL-A1* and *WPCL-D1* (Fig 4, panel (b)). The SHAQKYF amino acid motif is conserved in all LUX-family genes, and amino acid changes within the SHAQKYF motif potentially lead to loss-of-function in barley [27]. In addition, a 142 bp deletion in the 5′ UTR and the first exon, which could result in a truncated protein or a frameshift mutation, was detected in *WPCL-B1* of ‘Chogokuwase’ and ‘Geurumil’. The clock genes in ‘Chogokuwase’ and ‘Geurumil’ had arrhythmic expression patterns, whereas the *WPCL1* homoeologues were up-regulated (Fig 3). The *lux*/*pcl1* mutants in *Arabidopsis*, barley and einkorn wheat showed the same expression patterns of these clock genes [25, 27, 30]. Taken together, these findings strongly suggest that ‘Chogokuwase’ and ‘Geurumil’ contain loss-of-function natural variants of the *WPCL1* gene. Segregation analysis revealed that the homozygosity of recessive *wpcl1* alleles at all homoeoloci is a prerequisite for heading as early as ‘Chogokuwase’ (Figs 5 and 6). Segregation of F_2_s in the MG population suggested that *wpcl1* mutations are insufficient to explain the exceptionally early heading of ‘Chogokuwase’. A fraction of the segregants with the triple recessive *wpcl1* headed as late as those with a functional *WPCL1*, indicating the presence of another gene or genes that condition(s) the early heading phenotype. In the CN population, on the other hand, *wpcl1* mutations were sufficient to explain the earliness of ‘Chogokuwase’, suggesting that the hypothetical gene or genes is/are present in both ‘Chogokuwase’ and ‘Norin 61’ and are not segregating in the CN population. Alternatively, the number of days to heading may be unaffected by the hypothetical gene or genes in SD conditions.

### Loss-of-function mutations in *WPCL1* reduce photoperiod sensitivity by up-regulating *Ppd-1*

In wheat and barley, *Ppd-1*, the homologue of *Arabidopsis PRR7* and *PRR3*, has been regarded as the main regulator of photoperiod sensitivity [17, 42]. The photoperiod insensitive alleles of *Ppd-1* (*Ppd-1a*) invoke the mis-expression and/or increased expression of *Ppd-1* [20]. Thus, the pattern and level of *Ppd-1* expression are critical in regulating photoperiod sensitivity. The mutants of circadian clock components *lux*/*pcl1* and *elf3* in barley and wheat have increased levels of *Ppd-1* expression [24–28]. In *Arabidopsis*, LUX and ELF3 bind to the promoter of *PRR9* and repress its expression [30, 43]. Therefore, we hypothesized that WPCL1 might function as the repressor of *Ppd-1* (S2 Fig) [25]. In the present study, we found up-regulated expression of *Ppd-A1* and *Ppd-B1* in ‘Chogokuwase’ and ‘Geurumil’ where the functional *WPCL1* is absent (Fig 3). The expression pattern of *Ppd-B1* in ‘Chogokuwase’ was different from that in ‘Geurumil’. This difference might be reflecting the copy number variation at the *Ppd-B1* locus. Unlike *Ppd-A1* and *Ppd-B1*, there were no significant differences in expression patterns and levels of *Ppd-D1* transcripts between *wpcl1* mutants (‘Chogokuwase’ and ‘Geurumil’) and the cultivar with two functional *WPCL1* genes (‘Minaminokomugi’). All three cultivars carried the *Ppd-D1a* allele, which has a 2 kb deletion in the promoter region [33]. The 2 kb deletion overlaps with the positions of mutations in *Ppd-A1a* and *Ppd-B1a* [18–19, 21]. These observations suggest the 2 kb region, which includes a highly conserved ca. 100 bp sequence where putative *cis*-elements and regulatory motifs were predicted at the nucleotide sequence level, plays a critical role in *Ppd-1* regulation. We hypothesize that WPCL1, a member of the Myb transcription factor family, might bind to the promoter region of *Ppd-1* and repress *Ppd-1* expression. We will directly test this hypothesis in the future by conducting protein-DNA interaction assays such as chromatin immunoprecipitation experiments.

### *WPCL1* affects the vernalization requirement through *Vrn-2*

Day-neutral mutations such as *Ppd-D1a* and *eam8* increase the expression level of *Vrn-2* [39]. We confirmed that *wpcl1* mutations up-regulated *Vrn-2* expression (Fig 7). These observations suggested that photoperiodic signals were linked with the vernalization pathway via *Vrn-2* (S2 Fig). In fact, *Vrn-2* has a CCT domain that is found in proteins involved in light signal transduction [9]. ‘Geurumil’ is much more sensitive to vernalization than ‘Minaminokomugi’, although there was only a slight difference in the number of days to heading when plants were grown in the field where plants were fully vernalized (Table 1, Fig 1). The stronger vernalization requirement in ‘Geurumil’ is likely due to both up-regulation of *Vrn-2* and the absence of dominant *VRN-D1* alleles. Supporting evidence was reported in barley that overexpression of *Vrn-2* delayed heading and decreased the expression levels of *HvFT1* [44]. Therefore, the observed up-regulation of *Vrn-2* in *wpcl1* mutants could explain the delayed heading of ‘Geurumil’. We also showed that the absence of *Vrn-2* loci is crucial for early heading in the *wpcl1* mutants (Fig 8). ‘Chogokuwase’ exhibited vernalization-insensitive and early-heading phenotypes despite the up-regulated expression of *Vrn-2*. ‘Chogokuwase’ had higher *Vrn-1* expression that was due to the spring habit allele of *VRN-D1* (Fig 7). All of the early heading F_2_ plants in the MG population had the dominant *VRN-D1* allele (Fig 6). Our preceding experiment demonstrated that *Vrn-1* expression levels in leaves were associated with earliness [7]. Therefore, *VRN-D1* might be a candidate gene that confers early heading in ‘Chogokuwase’, a hypothesis that remains to be tested. Our observation that all the plants in early heading subgroup were homo- or heterozygous *VRN-D1* alleles (Fig 6, panel (b)) indicates that vernalization-insensivity is prerequisite for the ‘Chogokuwase’-type of early heading in the genetic backgrounds of the MG population. *Ppd-1* is known as a major gene controlling heading time in common wheat. Our study indicates that loss-of-function *wpcl1* accelerates heading time by up-regulating all *Ppd-1* homoeologues. Although *wpcl1* mutations could accelerate heading time, the effect of earliness is likely attenuated by *Vrn-2* up-regulation.

The timing of flowering is one of the most important traits for breeding, and early-heading cultivars are desired to avoid pre-harvest sprouting and associated poor grain quality. However, extra-early heading often reduces crop yield. In fact, ‘Chogokuwase’ is short and slender in appearance and the number of tillers is greatly reduced. In this study, we demonstrated that a loss-of-function mutation in *WPCL1* may accelerate and adjust flowering time due to the combination of vernalization gene activities in common wheat.

## Supporting Information

S1 Fig Copy number variation of the *Ppd-B1* sequences in seven cultivars. Relative mean values of PCR amplification levels of the *Ppd-B1* copy are shown with the standard deviation.
(TIF)

S2 Fig A revised model for the interaction of flowering-time genes in wheat. The role of *WPCL1*, whose orthologue in Arabidopsis forms the “Evening Complex” with *ELF3* [29], as a repressor of *Ppd-1* was added to the model proposed by Shimada et al. 2009 [13]. In their model, the triangle of *Vrn1–WFT–Vrn-2* gene interactions regulate the phase transition from vegetative to reproductive phase in wheat. *WFT* plays an integrative role in both the *WCO1*-related photoperiod pathway and the *VRN1*-related vernalization pathway. The expression levels of *Vrn-2* were up-regulated in ‘Chogokuwase’ that is triple homozygous for the loss-of-function alleles of *wpcl1*, indicating that *WPCL1* also repress *Vrn-2* directly and/or through promotion of *Ppd-1* expression (indicated by dashed lines).
(TIF)

S1 Table Primers used for genotyping of *Vrn-1*. (DOCX)

S2 Table Primers used for gene expression analyses. (DOCX)
