# Supplementary figures and images for: Loss-of-Function Mutations in Three Homoeologous PHYTOCLOCK 1 Genes in Common Wheat Are Associated with the Extra-Early Flowering Phenotype
Source: PLoS One. 2016 Oct 27;11(10):e0165618. doi: 10.1371/journal.pone.0165618 (PMC5082820; doi:10.1371/journal.pone.0165618)

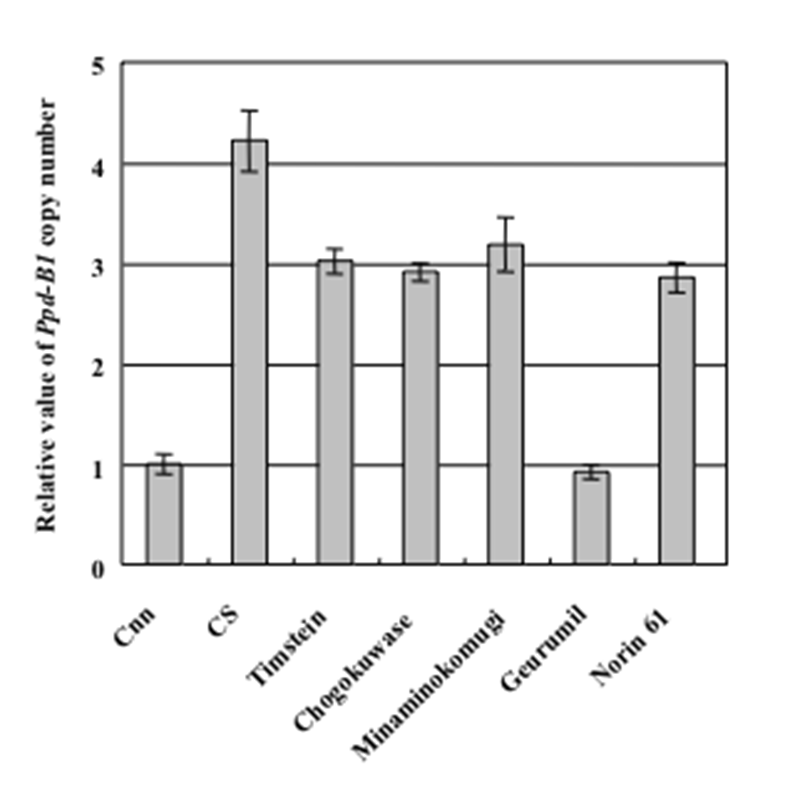

Supplement: S1 Fig — Relative mean values of PCR amplification levels of the Ppd-B1 copy are shown with the standard deviation. (TIF) [file pone.0165618.s001.tif]

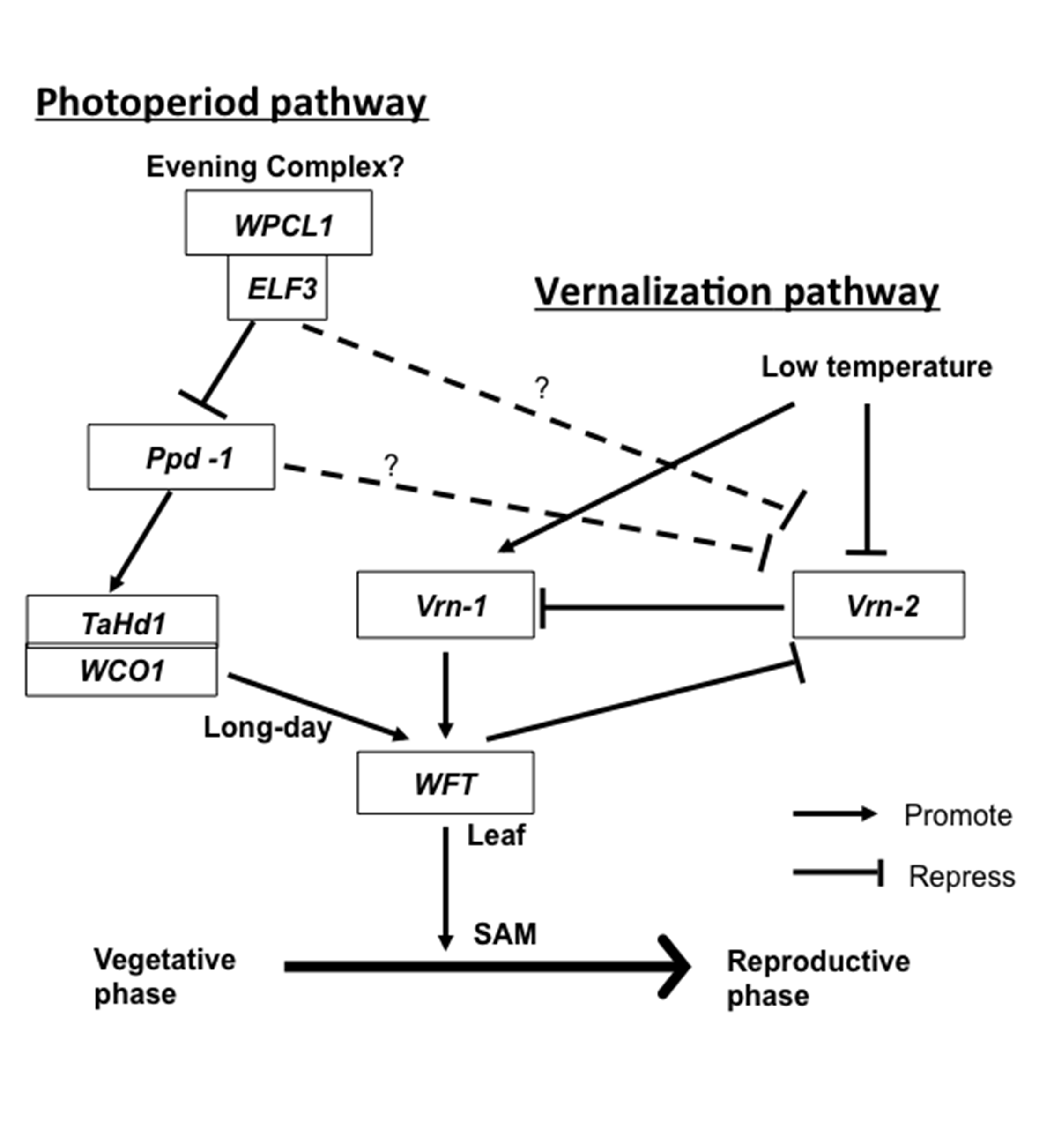

Supplement: S2 Fig — The role of WPCL1, whose orthologue in Arabidopsis forms the “Evening Complex” with ELF3 [29], as a repressor of Ppd-1 was added to the model proposed by Shimada et al. 2009 [13]. In their model, the triangle of Vrn1–WFT–Vrn-2 gene interactions regulate the phase transition from vegetative to reproductive phase in wheat. WFT plays an integrative role in both the WCO1-related photoperiod pathway and the VRN1-related vernalization pathway. The expression levels of Vrn-2 were up-regulated in ‘Chogokuwase’ that is triple homozygous for the loss-of-function alleles of wpcl1, indicating that WPCL1 also repress Vrn-2 directly and/or through promotion of Ppd-1 expression (indicated by dashed lines). (TIF) [file pone.0165618.s002.tif]
