# Supplementary material for: Loss-of-Function Mutations in Three Homoeologous PHYTOCLOCK 1 Genes in Common Wheat Are Associated with the Extra-Early Flowering Phenotype
Source: PLoS One. 2016 Oct 27;11(10):e0165618. doi: 10.1371/journal.pone.0165618 (PMC5082820; doi:10.1371/journal.pone.0165618)
Supplement: S1 Table — (DOCX) [file pone.0165618.s003.docx]

**S1 Table. Primers used for genotyping of *Vrn-1***

| **Allele** | **Forward name** | **Primer sequence (5′-3′)** | **Reverse name** | **Primer sequence (5′-3′)** |
| --- | --- | --- | --- | --- |
| *VRN-A1* | VRN1AF | GAAAGGAAAAATTCTGCTCG | VRN1R | TGCACCTTCCC(C/G)CGCCCCAT |
| *vrn-A1* | Intr1/C/F | GCACTCCTAACCCACTAACC | Intr1/AB/R | TCATCCATCATCAAGGCAAA |
| *VRN-B1* | Intr1/B/F | CAAGTGGAACGGTTAGGACA | Intr1/B/R3 | CTCATGCCAAAAATTGAAGATGA |
| *vrn-B1* | Intr1/B/F | CAAGTGGAACGGTTAGGACA | Intr1/B/R4 | CAAATGAAAAGGAATGAGAGCA |
| *VRN-D1* | Intr1/D/F | GTTGTCTGCCTCATCAAATCC | Intr1/D/R3 | GGTCACTGGTGGTCTGTGC |
| *vrn-D1* | Intr1/D/F | GTTGTCTGCCTCATCAAATCC | Intr1/D/R4 | AAATGAAAAGGAACGGAGCG |
