# Supplementary material for: Loss-of-Function Mutations in Three Homoeologous PHYTOCLOCK 1 Genes in Common Wheat Are Associated with the Extra-Early Flowering Phenotype
Source: PLoS One. 2016 Oct 27;11(10):e0165618. doi: 10.1371/journal.pone.0165618 (PMC5082820; doi:10.1371/journal.pone.0165618)
Supplement: S2 Table — (DOCX) [file pone.0165618.s004.docx]

**S2 Table. Primers used for gene expression analyses**

| **Gene** | **Forward sequence (5′-3′)** | **Reverse sequence (5′-3′)** | **Reference** |
| --- | --- | --- | --- |
| *Vrn-1* | GGAGAGGTCACTGCAGGAGGA | GCCGCTGGATGAATGCTG | Shimada et al. 2009 |
| *WFT* | GGTACAACTGGTGCCTCGTT | GTTGTAGAGCTCGGCGAAGT | Shimada et al. 2009 |
| *ZCCT1* | ATCACCTTCGCTGCTCTCTC | CCCACATCGTGCCATTTTAC | Distelfeld et al. 2009b |
| *ZCCT2* | CCACCATCGTGCCATTCT | CCCACCATCATCTCTGTATCAA | Distelfeld et al. 2009b |
| *WPCL-A1* | TCTCCTCCTACCCGCACTAC | GGATGGATCATGGACTGACTGA | this study |
| *WPCL-B1* | TCTCCTCCTACCCGCACTAC | GGATGGATCATGGACTGACTGA | this study |
| *WPCL-D1* | TCTCCTCCTACCCGCACTAC | GGATGGATCATGGACTGACTGA | this study |
| *TaTOC1* | GAGGATAGCTGAGTCATCTGC | GTCGGTCGCACCAGAGAAAT | AK333193 |
| *TaLHY* | ACGGCAGTTGCTCAGAAGTT | TCCTTCCCCACATCAATGGAA | HQ222606 |
| *Ppd-1A* | AGACAAGGCTGATGAAACGA | CGATGGATTGACCAAACTG | Shaw et al. 2012 |
| *Ppd-1B* | AAGACAAGGTTGATGACGTGA | GAGGGATTGATCACGTTGG | Shaw et al. 2012 |
| *Ppd-1D* | AAGACAAGGCTGATGAAATGAG | GAAGGATTGACCACATTGGA | Shaw et al. 2012 |
| *WCO1* | ATTGTGTTTCCAACGGCACC | AGAGGTACGCGGAGTCGG | Shimada et al. 2009 |
| *TaHd1* | GCAATGACATATCTTTCTCATCAA | AGAGCTCCATGGCTTCACT | Mizuno et al. 2012 |
| *TaGI* | TCTGGATTGCTCGAGATGAC | AAGCTTCACCGTCGACAA | Shaw et al. 2012 |
| *Actin* | GGCTGGTTTTGCTGGTGACGAAT | AATGAAGGAAGGCTGGAAGAGGA | Mizuno et al. 2010. |
